# Coexistence mechanism of ecological specialists and generalists based on a network dimension reduction method

**DOI:** 10.1002/ece3.10967

**Published:** 2024-02-21

**Authors:** Dongli Duan, Jiale Hang, Chengxing Wu, Xue Bai, Yisheng Rong, Gege Hou

**Affiliations:** ^1^ School of Information and Control Engineering Xi'an University of Architecture and Technology Xi'an China; ^2^ School of Economics NorthWest University of Politics and Law Xi'an China; ^3^ School of Mechanical Engineering Northwestern Polytechnical University Xi'an China

**Keywords:** coexistence mechanism, complex network, dimension reduction, dynamical networks

## Abstract

As an ecological strategy for species coexistence, some species adapt to a wide range of habitats, while others specialize in particular environments. Such ‘generalists’ and ‘specialists’ achieve normal ecological balance through a complex network of interactions between species. However, the role of these interactions in maintaining the coexistence of generalist and specialist species has not been elucidated within a general theoretical framework. Here, we analyze the ecological mechanism for the coexistence of specialist and generalist species in a class of mutualistic and competitive interaction ecosystems based on the network dimension reduction method. We find that ecological specialists and generalists can be identified based on the number of their respective interactions. We also find, using real‐world empirical network simulations, that the removal of ecological generalists can lead to the collapse of local ecosystems, which is rarely observed with the loss of ecological specialists.

## Graphical abstract

Provide a method to distinguish specialist and generalist species in ecosystems. Propose a dimension reduction method to obtain two‐dimensional models of specialists and generalists. Reveal that the decline of generalists may cause ecosystem collapse.

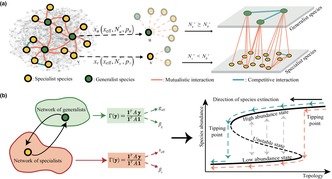

## 1 INTRODUCTION

In theoretical ecology, the study of the coexistence mechanism of habitat specialists and generalists has received high attention from researchers in the past few decades (Sriswasdi et al., 2017; Wilson & Yoshimura, 1994). The fitness of habitat specialists is restricted to certain environmental conditions, while generalists have a broader habitat tolerance. A number of factors are thought to influence the coexistence between specialists and generalists such as species' habitat selection (Mitchell et al., 2020; Morris, 1996), habitat fragmentation (Devictor et al., 2008; dos Anjos et al., 2019), and environmental heterogeneity (Kassen, 2002; Kneitel, 2018). These works provide useful insights into the coexistence mechanisms of habitat specialists and generalists. However, few studies have explored the impact of both positive and negative interactions between species on the coexistence mechanisms of habitat specialists and generalists (Nordberg & Schwarzkopf, 2019).

Species in ecosystems rarely live in isolation, but depend on different types of interactions to coexist with other species, such as trophic, competitive, and mutualistic interactions (Godoy, 2019). The combined effects of different interactions between species have been shown to play an important role in, but not limited to, maintaining ecosystem stability (Rohr et al., 2014; Thébault & Fontaine, 2010; Xu et al., 2023), species diversity (Losapio et al., 2021; Pascual‐García & Bastolla, 2017), co‐evolution (Bronstein, 2009; Guimaraes et al., 2017), and species invasion (Duan et al., 2022; Legault et al., 2020). The study of the coexistence of specialist and generalist species under different types of interactions can not only help to understand the universal characteristics of species interactions but also significantly improve our understanding of the division of ecological functions among species (Pasquaretta & Jeanson, 2018). However, the real ecosystem is a multidimensional system containing a large number of specialists and generalists and complex interactions between species, and many mathematical tools for analyzing coexistence mechanisms of species are valid for low‐dimensional systems (Levine et al., 2017; Singh & Baruah, 2021). Therefore, it is logically challenging to empirically analyze coexistence mechanisms between habitat specialists and generalists.

Dimension reduction theory based on complex network science provides an effective approach to solve this problem. Gao et al. (2016) firstly proposed a dimension reduction framework to identify the control and behavioral parameters of a N‐dimensional complex system using input and output degrees as weights and to derive a general one‐dimensional (1D) dynamic function. Jiang et al. (2018) then performed an effective 2D dimension reduction using the degrees and eigenvector respectively as weights and showed that weighted averaging during the dimension reduction process is necessary due to the lack of sufficient randomness in the real network structure. At present, dimension reduction has been widely applied to various complex systems (Dongli et al., 2021; Laurence et al., 2019).

In this paper, we develop an analytical framework based on dimension reduction (Gao et al., 2016; Jiang et al., 2018) to study interaction networks with specialists and generalists and to find fundamental characteristics that emerge from habitat specialists and generalists under both mutualistic and competitive interactions. We first reduce the dimension of the local ecosystem and obtain a one‐dimensional representation of the dynamical model of individual species. By analyzing the dynamics of individual species, we develop a method to identify habitat specialists and generalists through mutualistic links. We then construct a set of two nonlinear functions for habitat specialists and generalists to capture the fundamental parameters of the original network. Using real plant‐pollinator networks, we further study the composition of specialists and generalists and analyze the impact of disturbance on the subset network of specialists and generalists. Our results suggest that the concepts of specialists and generalists are indispensable for understanding the composition and functioning of ecosystems.

## 2 MODEL AND METHODS

### 2.1 Ecosystem dynamics model involving both mutualistic and competitive interactions

We extended a complex network model (Gao et al., 2016; Holland et al., 2002) describing mutualistic interactions between plants and pollinators to include competitive interactions between species (Dongli et al., 2022; Duan et al., 2022):

(1) $$\frac{{\mathrm{dx}}_{i}}{\mathrm{dt}}=B_{i}+x_{i}\left(1-\frac{x_{i}}{K_{i}}\right)\left(\frac{x_{i}}{C_{i}}-1\right)+\sum_{j=1}^{N}\frac{M_{\mathrm{ij}}^{+}x_{i}x_{j}}{D_{i}+E_{i}x_{i}+H_{j}x_{j}}-\sum_{j=1}^{N}\frac{M_{\mathrm{ij}}^{-}x_{i}x_{j}}{1+F_{j}x_{j}},$$

where $x_{i}$ is the population of the ith species, $B_{i}$ is the migration rate from neighboring ecosystems, $K_{i}$ (Tsoularis & Wallace, 2002) measures the ecosystem carrying capacity, and $C_{i}$ (Courchamp et al., 1999) represents the Allee effect constant. Adjacency matrix $M_{\mathrm{ij}}^{+}$ mutualistic interactions between species, and $D_{i}$, $E_{i}$ and $H_{j}$ control the saturation rate of the response function. In addition, we consider the competitive interaction between species in the fourth term on the right side of the above equation. This describes a scenario with Holling type 2 functional response (Dawes & Souza, 2013): $x_{i}$ initially decreases rapidly with increasing competitors' density, but then levels off with further increase in competitors' density. $M_{\mathrm{ij}}^{-}$ captures the competitive interaction between species i and j, and $F_{j}$ is characteristic coefficient.

### 2.2 Collective abundances of ecosystems and one‐dimensional representation of individual species

Equation (1) is a typical high‐dimensional dynamical equation, which is difficult to analyze especially when the scale is large enough. One effective approach is to collapse Equation (1) into a one‐dimensional version. In this work, we consider dimension reduction formalism for N‐dimensional representation using degree‐based weighting method (Gao et al., 2016; Jiang et al., 2018). The degree‐weighted method uses the input and output degrees of nodes as weights to map the average abundance of the system and is applied to the mutualistic network (Gao et al., 2016). Next, we use the degree‐weighted method to reduce the dimension of a class of coupled networks containing mutualistic and competitive interactions.

To directly encode multiple interactions, we first introduce the matrix $A_{\mathrm{ij}}$ to couple the interaction of $M_{\mathrm{ij}}^{+}$ and $M_{\mathrm{ij}}^{-}$. If the interaction between i and j is mutually beneficial $A_{\mathrm{ij}}=1$; if the interaction between i and j is competitive $A_{\mathrm{ij}}=-1$; otherwise $A_{\mathrm{ij}}=0$. Then, we define the competition strength p as the ratio of the sum of the competitive links to the sum of all link in the bipartite matrix $A_{\mathrm{ij}}$, that is, $p=\sum_{i=1}^{N}\sum_{j=1}^{N}\frac{|M_{\mathrm{ij}}^{-}|}{|A_{\mathrm{ij}}|}$.

Let us introduce an operator $\Gamma \left(y\right)$ to describe the global state of the system by capturing the instantaneous state of all nearest nodes. The operator $\Gamma \left(y\right)$ can be written as (Gao et al., 2016)

(2) $$\begin{array}{l} \Gamma \left(y\right)=\frac{1^{T}Ay}{1^{T}A1}=\frac{\sum_{i=1}^{N}\sum_{j=1}^{N}A_{\mathrm{ij}}y_{j}}{\sum_{i=1}^{N}\sum_{j=1}^{N}A_{\mathrm{ij}}}=\frac{\frac{1}{N}\sum_{j=1}^{N}S_{j}^{\text{out}}y_{j}}{\frac{1}{N}\sum_{j=1}^{N}S_{j}^{\text{out}}}=\frac{<S_{j}^{\text{out}}y_{j}>}{<S_{j}^{\text{out}}>}, \end{array}$$

where 1 is the unit vector $1={\left(1,\ldots ,1\right)}^{T}$, y is the state vector $y={\left[y_{1},y_{2},\ldots ,y_{N}\right]}^{T}$, and $S_{j}^{\text{out}}$ is the outgoing weighted degree of node j in the bipartite matrix $A_{\mathrm{ij}}$ $\left(S_{j}^{\text{out}},=,\sum_{i=1}^{N},|,A_{\mathrm{ij}},\text{∣}\right)$.

Let $g_{j}\left(x_{i}\right)=\frac{x_{i}x_{j}}{D+{\mathrm{Ex}}_{i}+{\mathrm{Hx}}_{j}}$, we use $\Gamma \left(g_{i}\left(x_{i},x\right)\right)$ to capture the effective influence obtained by all species through mutualistic interactions. The third term on the right side of Equation (1) is transformed as

(3) $$\sum_{j=1}^{N}M_{\mathrm{ij}}^{+}\frac{x_{i}x_{j}}{D+{\mathrm{Ex}}_{i}+{\mathrm{Hx}}_{j}}=\left(1-p\right)S_{i}^{\text{in}}\Gamma \left(g_{i}\left(x_{i},x\right)\right).$$

where $S_{i}^{\text{in}}$ is the incoming weighted degree of node i in the bipartite matrix $A_{\mathrm{ij}}$ $\left(S_{i}^{\text{in}},=,\sum_{j=1}^{N},|,A_{\mathrm{ij}},\text{∣}\right)$, $x={\left[x_{1},x_{2},\ldots ,x_{N}\right]}^{T}$ and $g_{i}={\left[\frac{x_{i}x_{1}}{D+{\mathrm{Ex}}_{i}+{\mathrm{Hx}}_{1}},\frac{x_{i}x_{2}}{D+{\mathrm{Ex}}_{i}+{\mathrm{Hx}}_{2}},\ldots ,\frac{x_{i}x_{N}}{D+{\mathrm{Ex}}_{i}+{\mathrm{Hx}}_{N}}\right]}^{T}$. We assume that competitive links in the ecosystem are equally distributed across interactions of each species. Similarly, making $h_{j}\left(x_{i}\right)=\frac{x_{i}x_{j}}{1+{\mathrm{Fx}}_{j}}$, the fourth term on the right part of Equation (1) can be rewritten as

(4) $$\sum_{j=1}^{N}M_{\mathrm{ij}}^{-}\frac{x_{i}x_{j}}{1+{\mathrm{Fx}}_{j}}={\mathrm{pS}}_{i}^{\text{in}}\Gamma \left(h_{i}\left(x_{i},x\right)\right).$$

where $h_{i}={\left[\frac{x_{i}x_{1}}{1+{\mathrm{Fx}}_{1}},\frac{x_{i}x_{2}}{1+{\mathrm{Fx}}_{2}},\ldots ,\frac{x_{i}x_{N}}{1+{\mathrm{Fx}}_{N}}\right]}^{T}$.

Considering Equations (3) and (4), we can rewrite Equation (1) as

(5) $$\begin{array}{l} \frac{{\mathrm{dx}}_{i}}{\mathrm{dt}}=B+x_{i}\left(1-\frac{x_{i}}{K}\right)\left(\frac{x_{i}}{C}-1\right)+\left(1-p\right)S_{i}^{\text{in}}\Gamma \left(g_{i}\left(x_{i},x\right)\right)-{\mathrm{pS}}_{i}^{\text{in}}\Gamma \left(h_{i}\left(x_{i},x\right)\right). \end{array}$$

In Equation (5), the operator $\Gamma \left(y\right)$ provides the average influence that species i received from all its nearest neighbors j. Then, we apply the Hadamard product (Horn, 1990) and mean field theory (Gao et al., 2016) to Equation (5). We further obtain a one‐dimensional representation of the N‐dimensional system

(6) $$\begin{array}{l} \frac{{\mathrm{dx}}_{\mathrm{eff}}}{\mathrm{dt}}=B+x_{\mathrm{eff}}\left(1-\frac{x_{\mathrm{eff}}}{K}\right)\left(\frac{x_{\mathrm{eff}}}{C}-1\right)+\frac{\left(1-p\right)\beta_{\mathrm{eff}}x_{\mathrm{eff}}^{2}}{D+\left(E+H\right)x_{\mathrm{eff}}}-\frac{p\beta_{\mathrm{eff}}x_{\mathrm{eff}}^{2}}{1+{\mathrm{Fx}}_{\mathrm{eff}}}, \end{array}$$

where $x_{\mathrm{eff}}$ is the nearest neighbor weighted average abundance ($x_{\mathrm{eff}}=\Gamma \left(x\right)=\frac{1^{T}Ax}{1^{T}A1}=\frac{\left\langle S^{\text{out}}x\right\rangle }{\left\langle S\right\rangle }$) and $\beta_{\mathrm{eff}}$ is the nearest neighbor weighted average degree ($\beta_{\mathrm{eff}}=\Gamma \left(S^{\text{in}}\right)=\frac{1^{T}{\mathrm{AS}}^{\text{in}}}{1^{T}A1}=\frac{\left\langle S^{\text{out}}S^{\text{in}}\right\rangle }{\left\langle S\right\rangle }$).

We get the collective abundance of the system as Equation (6). Next, we further obtain the one‐dimensional representation of the dynamics of individual species. The abundance of species i is affected by the interaction of species j. Instead of studying the role of each species j on i, we used the collective abundance $x_{\mathrm{eff}}$ to capture the effect of species j. Equation (1) can be written as

(7) $$\begin{array}{l} \frac{{\mathrm{dx}}_{i}}{\mathrm{dt}}=B_{i}+x_{i}\left(1-\frac{x_{i}}{K_{i}}\right)\left(\frac{x_{i}}{C_{i}}-1\right)+\frac{N_{i}^{+}x_{i}x_{\mathrm{eff}}}{D_{i}+E_{i}x_{i}+H_{\mathrm{eff}}x_{\mathrm{eff}}}-\frac{N_{i}^{+}p_{i}x_{i}x_{\mathrm{eff}}}{\left(1-p_{i}\right)\left(1+F_{\mathrm{eff}}x_{\mathrm{eff}}\right)}. \end{array}$$

where $p_{i}$ is the competitive intensity of ith species ($p_{i}=\frac{N_{i}^{-}}{N_{i}^{+}+N_{i}^{-}}$), $N_{i}^{+}$ is the sum of the mutualistic interactions of the species i, and $N_{i}^{-}$ is the sum of the competitive interactions of the species i. $H_{\mathrm{eff}}$ and $F_{\mathrm{eff}}$ are obtained through the operators $\Gamma \left(H\right)$ and $\Gamma \left(F\right)$, respectively.

Ultimately, we use the collective abundance of the system to derive effective one‐dimensional equation in the form of Equation (6) and obtain a one‐dimensional representation of individual species Equation (7). In this way, not only the mathematical analysis can be conveniently performed but also the effective characteristics of the system can be obtained.

### 2.3 Two‐dimensional reduced model of specialist and generalist species

**FIGURE 1:**
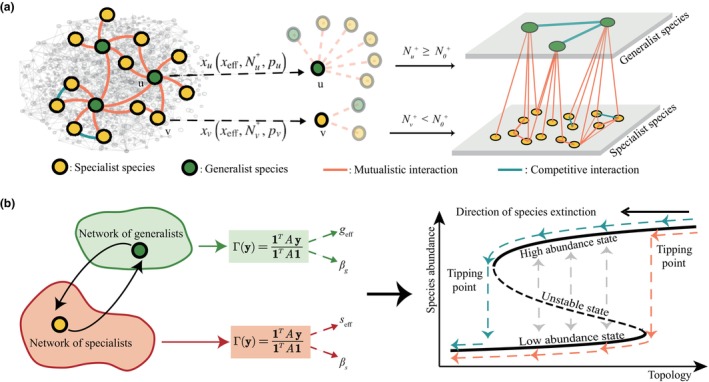
Schematic diagram of modeling and dimension reduction of the two‐layer network composed of habitat generalists and specialists. (a) The local ecosystem is modeled as a two‐layer network of generalists and specialists. $x_{\mathrm{eff}}$ describes the collective state of a local ecosystem, $N_{i}^{+}$ captures the mutualistic links of individual species, and $p_{i}$ is the competition intensity of species. The characteristics of individual species can be written as a function of $x_{\mathrm{eff}}$, $N_{i}^{+}$ and $p_{i}$. $N_{0}^{+}$ is the threshold that distinguishes specialists from generalists, when $N_{i}^{+}\geq N_{0}^{+}$ species are generalists and $N_{i}^{+}<N_{0}^{+}$ are specialists. (b) Two‐dimensional reduction of multi‐dimensional local ecosystem. With the help of operator $\Gamma \left(y\right)$, we reduce the dimensions of the two‐layer network and obtain the one‐dimensional equations of ecological generalists and specialists respectively. $g_{\mathrm{eff}}$ and $s_{\mathrm{eff}}$ capture the collective average abundances of ecological generalists and specialists, respectively. $\beta_{g}$ and $\beta_{s}$ represent the weighted average degree of ecological generalists and specialists, respectively. In this low‐dimensional system, we can analyze the state of the specialist and generalist.

Ecosystems can be viewed as a two‐layer network of specialist and generalist species. As shown in Figure 1a, a local ecosystem is partitioned into a two‐layer network of specialists and generalists. We abstract the mathematical equations of generalists and specialists' abundances from Equation (1) as

(8) $$\begin{array}{l} \frac{{\mathrm{dg}}_{i}}{\mathrm{dt}}=B_{i}+g_{i}\left(1-\frac{g_{i}}{K_{i}}\right)\left(\frac{g_{i}}{C_{i}}-1\right)+\sum_{j=1}^{N}G_{\mathrm{ij}}^{+}\frac{g_{i}\gamma_{j}}{D_{i}+E_{i}g_{i}+H_{j}\gamma_{j}}-\sum_{j=1}^{N}G_{\mathrm{ij}}^{-}\frac{g_{i}\gamma_{j}}{1+F_{j}\gamma_{j}} \end{array}$$

and

(9) $$\begin{array}{l} \frac{{\mathrm{ds}}_{i}}{\mathrm{dt}}=B_{i}+s_{i}\left(1-\frac{s_{i}}{K_{i}}\right)\left(\frac{s_{i}}{C_{i}}-1\right)+\sum_{j=1}^{N}S_{\mathrm{ij}}^{+}\frac{s_{i}\gamma_{j}}{D_{i}+E_{i}s_{i}+H_{j}\gamma_{j}}-\sum_{j=1}^{N}S_{\mathrm{ij}}^{-}\frac{s_{i}\gamma_{j}}{1+F_{j}\gamma_{j}} \end{array},$$

where $G_{\mathrm{ij}}^{+}$ and $S_{\mathrm{ij}}^{+}$ control the mutualistic interaction received by the generalist and specialist, respectively. $G_{\mathrm{ij}}^{-}$ and $S_{\mathrm{ij}}^{-}$ capture the competitive interaction received by the generalist and specialist, respectively. $\gamma_{j}$ is a state variable that represents the abundance of species j. If j is a generalist species, $\gamma_{j}$ is represented as the abundance of the generalist species $g_{j}$. Otherwise, $\gamma_{j}$ represents the abundance of the specialist species $s_{j}$. Similar to Section 2.2, we then use dimension reduction to capture the collective abundances of generalists and specialists using $g_{\mathrm{eff}}$ and $s_{\mathrm{eff}}$, respectively (Figure 1b). This allows us to reduce the dimension of Equations (8) and (9) and obtain efficient low‐dimensional representations as
(10)

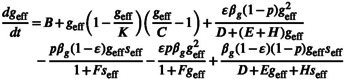

and
(11)

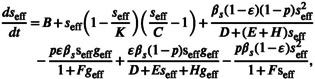

where $g_{\mathrm{eff}}$ and $s_{\mathrm{eff}}$ are the collective average abundances of ecological generalists and specialists, respectively. $\beta_{g}$ and $\beta_{s}$ describe the nearest neighbor weighted average degree of generalists and specialists, respectively. ε captures the proportion of generalist species in the local ecosystem.

Above, we obtained the two‐dimensional reduced model of specialists and generalists. Equations (8) and (9) can be solved for simulated solutions of generalist and specialist species, respectively. Equations (10) and (11) can be solved for analytical solutions of generalist and specialist, respectively. The parameters used in all the work of this paper are as follows 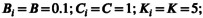 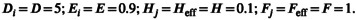


### 2.4 Databases of plant‐pollinator networks

We assembled some real plant‐pollinator networks from the web‐of‐life database (www.web‐of‐life.es). As shown in Table 1, these plant‐pollinator networks span a broad geographical range with different structures and species numbers. However, these plant‐pollinator networks only contain mutualistic relationships and do not include competitive relationships between species.

**TABLE 1:** Plant‐pollinator networks.

ID	Species	Interactions	Location
M_PL_001 (Arroyo et al., 1985)	185	361	Chile
M_PL_002 (Arroyo et al., 1985)	107	196	Chile
M_PL_008 (Dupont et al., 2003)	49	106	Canary Islands
M_PL_010 (Fortuna et al., 2014 )	107	456	Zackenberg
M_PL_018 (Fortuna et al., 2014)	144	383	Denmark
M_PL_019 (Inouye & Pyke, 1988)	125	264	Australia
M_PL_030 (Ramirez & Brito, 1992)	81	109	Venezuela
M_PL_031 (Ramirez, 1989)	97	156	Venezuela
M_PL_033 (Small, 1976)	47	141	Canada
M_PL_041 (Hung et al., 2018)	74	145	Dominica
M_PL_043 (Jiang et al., 2018)	110	250	Denmark
M_PL_058 (Bartomeus et al., 2008)	113	319	Spain

We considered a well‐studied approach to construct the new network involving mutualism and competition through plant‐pollination network, in which species in the same group are competitive for resources (e.g. food, space) and provide food for other groups which is mutualistic species of it (Gracia‐Lázaro et al., 2018). We assumed that the strength of the interaction between species are equal, $A_{\mathrm{ij}}=1$ indicates the mutualistic relationship between species i and j, and $A_{\mathrm{ij}}=-1$ represents the competitive relationship between species i and j, otherwise $A_{\mathrm{ij}}=0$.

## 3 RESULTS

### 3.1 Distinguish ecological specialists and generalists through mutualistic interactions

**FIGURE 2:**
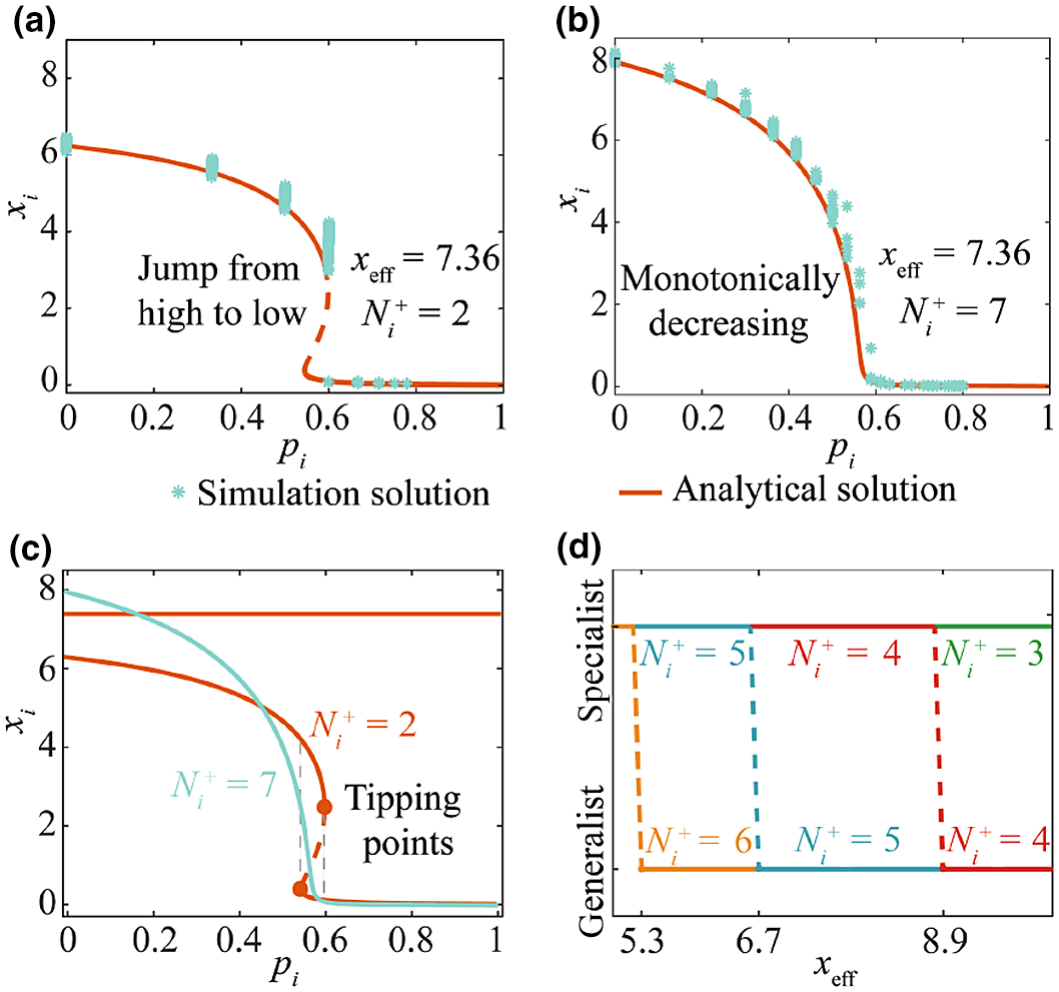
Ecological specialists and generalists can be distinguished through mutualistic interactions. $x_{i}$ captures the abundance of species i, $p_{i}$ describes the competition strength of species i, $N_{i}^{+}$ is the number of mutualistic links of species i, and $x_{\mathrm{eff}}$ represents the collective state of local ecosystems. (a, b) Congruence of analytical and simulation solutions for individual species when $N_{i}^{+}$ is 2 and 7, respectively. The analytical solution is solved by Equation (7). We simulate individual species with different competition intensities using the real ecosystem M_PL_002 (see Section 2.2 for details). ODE45 is used to solve the simulation solution of individual species. From 4000 simulations of individual species, we present results when $x_{\mathrm{eff}}=7.36$. (c) Distinguish between habitat specialists and generalists based on differences in species dynamics brought about by mutualistic links: specialists have tipping points. (d) We solve the range of mutualistic links between specialists and generalists in different ecosystems. For example, for local ecosystems with $x_{\mathrm{eff}}$ between 6.7 and 8.9, the mutualistic links for habitat specialists are at most 4, while the mutualistic links for habitat generalists are $N_{i}^{+}\geq 5$.

The abundance of a species is influenced by the interaction of its associated species. We replaced the role of these associated species with the weighted average neighborhood abundance $x_{\mathrm{eff}}$ and obtained Equation (7) which describes the abundance of individual species in Section 2.2. We formalized Equation (7) as shown in Figure 2c. We found that the abundance of species with more mutualistic links changed continuously from high to low with the increase in competition intensity, while the species with fewer mutualistic links jumped from high to low abundance. In other words, species with more mutualistic links are significantly different from species with only a few mutualistic links in local ecosystems.

To verify this results, we simulated the interaction of individual species using the real ecosystem M_PL_002 as the original network. We set different competition intensities to simulate various survival conditions of species. We first introduced a new node with a fixed number of mutualistic links into the original network. Mutualistic links are randomly linked with nodes in the original network. The introduced nodes are then set to generate competing links with nodes in the original network with a probability ranging from 0 to 1. Each probability was simulated 200 times. The step size of the probability change is 0.05. The results of the analytical solution and the simulation solution are shown in Figure 2a,b, indicating that the species with fewer and more mutualistic links had different patterns.

Ecological generalists interact with a wide range of species, while ecological specialists interact only with specific species. Based on the fact that different species of the reciprocal link have different phase transition patterns, we divided species in the ecosystem into generalists and specialists. For each type of local ecosystem, we calculated the mutualistic link between habitat specialists and generalists based on the number of species tipping points (Figure 2d). More specifically, specialist have two tipping points, while generalist have only one.

### 3.2 Predictive power and analytical analysis of reducted models for generalists and specialists

Ecosystems, consisting of thousands of species, can be described as a two‐layer network of specialists and generalists. We reduced the dimension of this two‐layer network to arrive a two‐dimensional model in Section 2.3. Equations (10) and (11) describe reducted models of specialist and generalist species, respectively.

**FIGURE 3:**
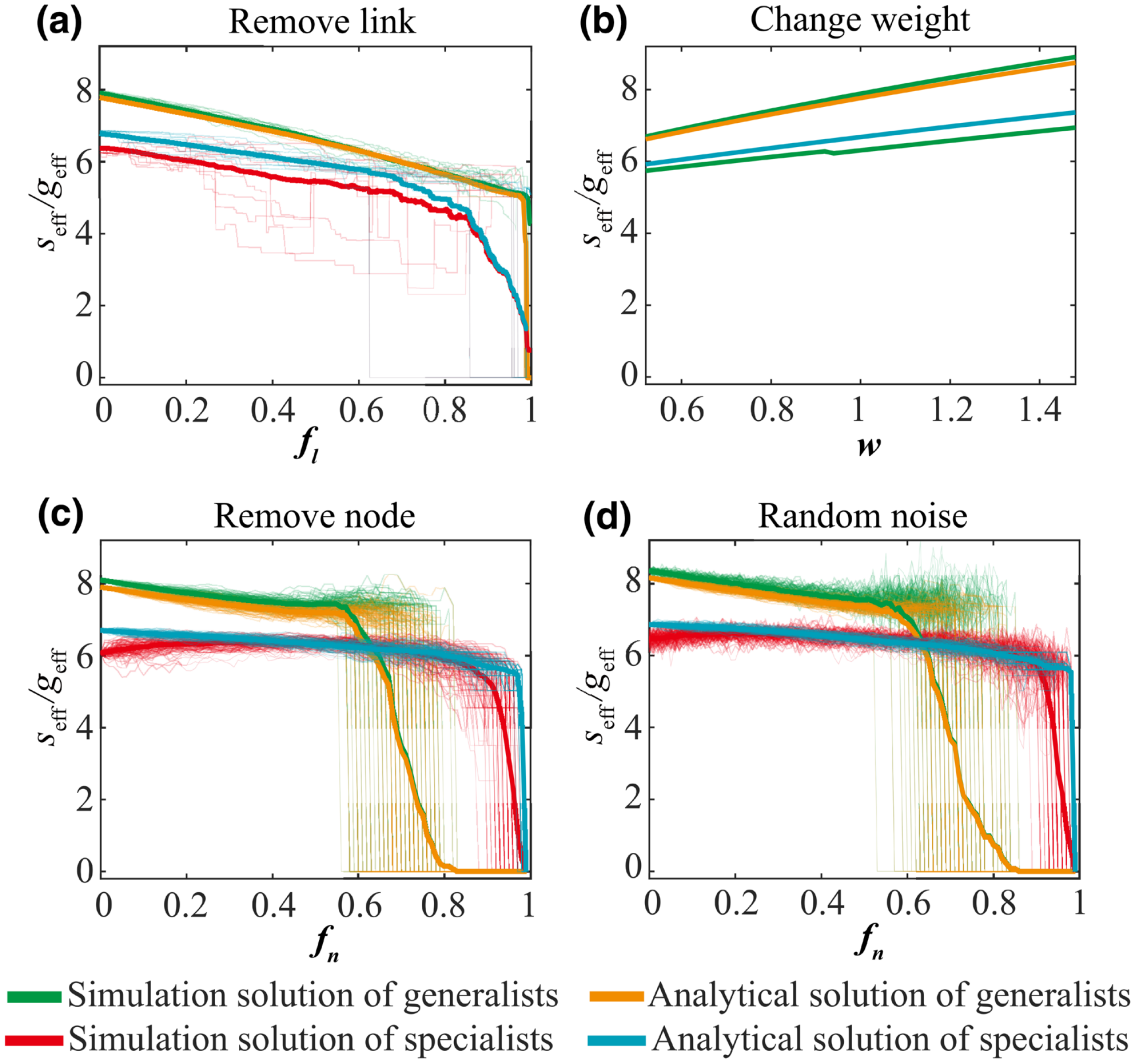
The predictive power of reduced model of generalists and specialists in the presence of disturbances. Using the ER (Erdos‐Renyi) model, we generate the simulation network of 100 nodes. $g_{\mathrm{eff}}$ and $s_{\mathrm{eff}}$ are used to capture the collective abundances of the generalist and specialist, respectively. Specialists and generalists in the network are identified using the method in Figure 1d. The simulation solution is solved by Equations (8) and (9), respectively. The analytical solution is solved by Equations (10) and (11), respectively. Each type of disturbance is simulated 100 times (thin line). The thick solid line is the average of the results of 100 simulations. (a) Randomly remove links of nodes. $f_{l}$ is the proportion of links removed from the entire network. (b) Change the weight of the link. We set the weighting factor w and change the weight of the link in the network by $A\to w^{*}A$. (c) Simulate species extinction by removing nodes. $f_{n}$ is the proportion of nodes deleted. (d) The effect of noise on the predictive ability of the reduced model. We simulate species extinction and add independent white Gaussian noise to the dynamics equation of each species. The noise obeys the normal distribution with zero mean and 0.5 variance.

To verify that Equations (10) and (11) capture the fundamental structure and behavior of the original system, we tested the consistency of the analytical solution and the simulation solution under disturbances. We used ER (Erdos‐Renyi) network to simulate the local ecosystem. The disturbances are modeled as removing links, changing weights, removing nodes, and random noise, respectively. The results are shown in Figure 3. We can see that the analytical solution effectively captures the collective abundances of specialists and generalists even when different types of disturbances occur.

### 3.3 The compositional characteristics of generalists and specialists in real‐world ecosystems

**FIGURE 4:**
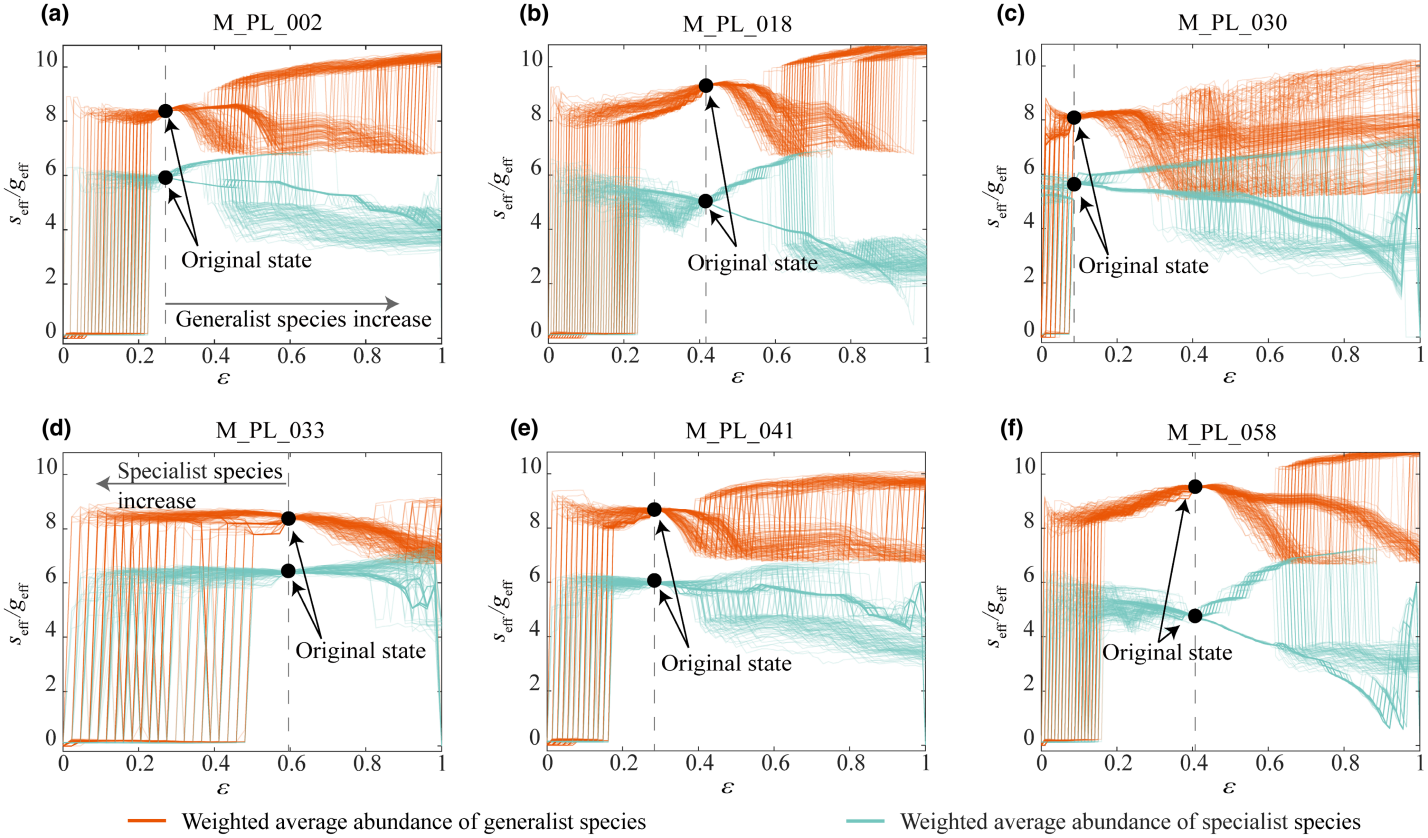
Composition characteristics of specialists and generalists in real ecosystems. We use the real plant‐pollination network in Table 1 and calculate its composition of generalists and specialists. The black circles are the original composition of specialists and generalists in the real network. We simulate the change of species in local ecosystems: generalist species randomly remove links to become specialists; specialist species become generalists by randomly adding links. ε is the proportion of generalists in the local ecosystem. The collective abundances of generalists and specialist's species are calculated separately in the change process. Each change state is simulated 100 times. The composition of specialists and generalists in the network is recalculated if species changes. (a) Species composition and simulations of species change in M_PL_002. (b–f) Similar results are observed in other real ecosystems.

To further investigate ecological composition characteristics between generalists and specialists, we analyzed the ecological networks from the real world in Table 1. We first calculated the composition and collective abundances of specialist and generalist species of these networks. The results are shown in Figure 4, the original state for the composition and collective abundance distribution of specialist and generalist species of these networks.

We then varied the composition of generalists and specialists in these networks by simulating the conversion between specialists and generalists. The species change process is simulated by generalists randomly removing links into specialists and specialists randomly adding links into generalists. We calculated the weighted average abundance of specialists and generalists in the change process separately. As shown in Figure 4, we observed that as the number of generalists in the ecosystem decreases, it may lead to the collapse of the local ecosystem, while the increase of generalists rarely leads to collapse. In other words, as the proportion of generalist species increases, the ecosystem maintains its original state of high collective abundance. However, when the proportion of specialist species in the local ecosystem increases, the system is at risk of collapse. Generalists are the core component of ecosystems. The decline of generalists leads to the loss of numerous interactions in local ecosystems, which can lead to ecosystem collapse.

### 3.4 The collective abundance of the subset network of specialists and generalists in the presence of disturbances

In contrast to the global network, we divided the local ecosystem into the subset network consisting of specialists and generalists. We compared the global network and the subset of networks of specialists and generalists in the presence of random disturbances. The $x_{\mathrm{eff}}$, $s_{\mathrm{eff}}$, and $g_{\mathrm{eff}}$ are used to capture the collective abundance of the global network, specialists, and generalists, respectively. Random disturbances are modeled as species extinctions. The real plant‐pollinator networks in Table 1 are used as the original network. At each time step, extinction events are simulated by removing nodes in the global network.

**FIGURE 5:**
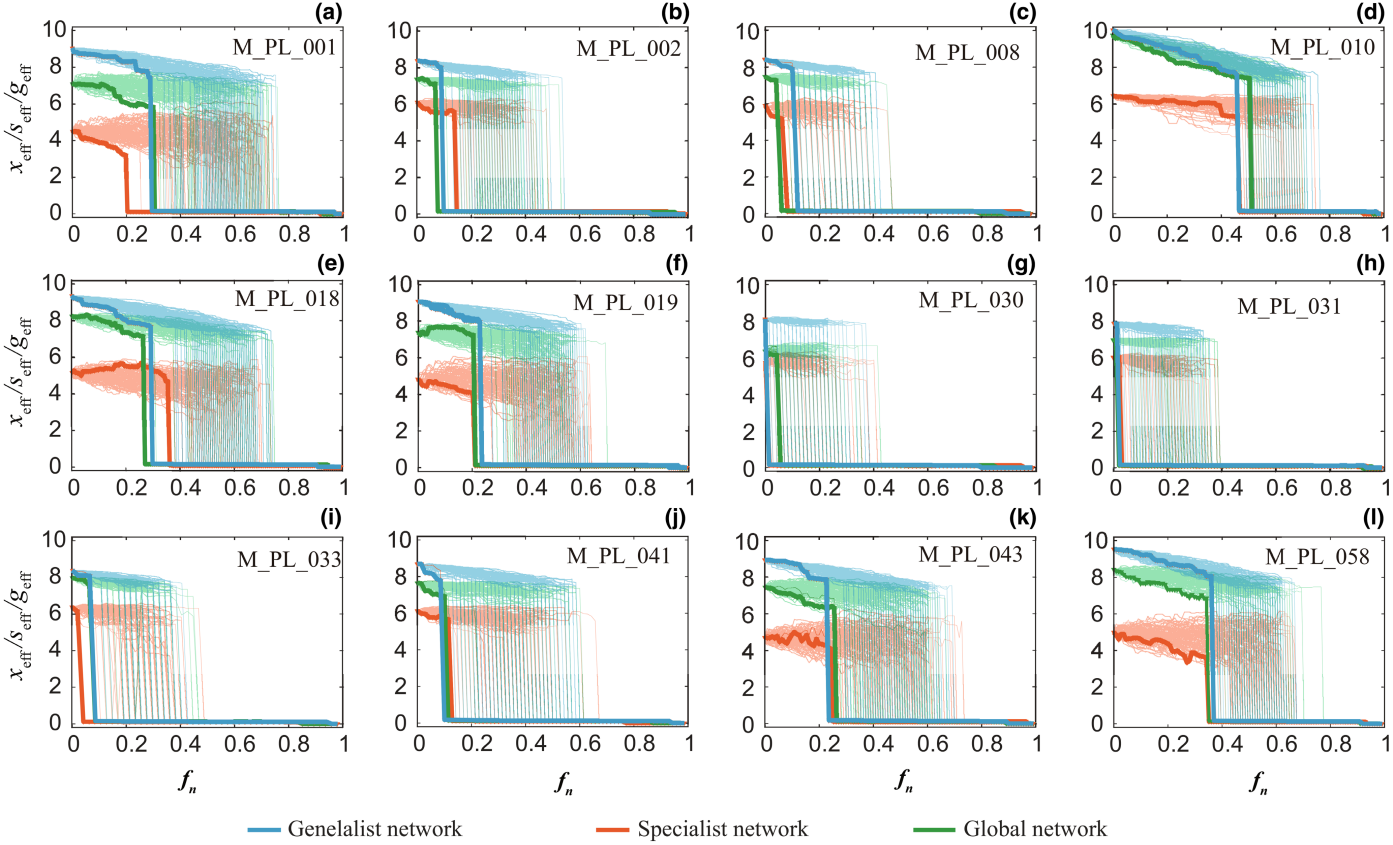
The collective abundance in the subset network of specialists and generalists. We simulate the extinction of species in the ecosystem. The extinction of species in the ecosystem may correspond to removing one or more nodes in the global network. The removed nodes are randomly selected in the global network. Specialists and generalists in the network are identified using the method in Figure 2d. The $g_{\mathrm{eff}}$, $s_{\mathrm{eff}}$ and $x_{\mathrm{eff}}$ are calculated separately during node removal. $f_{n}$ is the proportion of species extinction in the global network. Each network is simulated 100 times. (a) The average weighted abundance of generalists, specialists, and global network in M_PL_001. Both the subset network and the global network collapse from high abundance state to an undesired low abundance state. We highlight the first of these realizations with bold lines. (b–l) Ecosystems collapse from high‐abundance to low‐abundance state behavior has been observed in other networks.

The results are shown in Figure 5, and we can see that as the number of extinct species increases, there is a tipping point in the ecosystem where the abundance of species drops to almost zero. Our analysis of 12 real networks and found that the range of tipping points is highly unpredictable. We also found that the subset networks of specialists and generalists collapse before the global network, as shown in Figure 5a,d,k, and, in a couple of cases, the global network collapses before the other two subset networks.

## 4 CONCLUSIONS AND DISCUSSION

Disentangling the mechanisms that maintain the coexistence of habitat species is a central topic of ecological research. In this paper, we investigated the coexistence mechanisms of specialist and generalist species under both mutualistic and competitive interactions. To capture the effective abundance of habitat specialists and generalists, we used a degree‐based weighting method to reduce the dimension of the two‐layer network to obtain a 2D reduced model of generalists and specialists. Our simulation and analytical results show that our 2D reduced model can capture the structural and behavioral characteristics of the two‐layer network. In addition, we applied dimension reduction to capture the overall abundance of nearby ecosystems and obtain one‐dimensional representations of individual species (see Section 2.2 for details). Significantly, many different efficient methods (e.g. spectral weighted method) have recently been proposed to map multidimensional systems to low‐dimensional states (Duan et al., 2021; Jiang et al., 2018; Laurence et al., 2019; Wu et al., 2023), and applying these methods to our work may yield unexpected benefits.

Our work provided insight into the role of mutualistic and competitive interactions in ecosystems and found that species with different mutualistic links have different characteristics. Specifically, as competition for species increases, the abundance of species with fewer mutualistic links collapses from a high abundance state to an extinction state, while the abundance of species with more mutualistic links decreases monotonically (Figure 2). In this paper, we used this species diversity to identify generalist and specialist species in local ecosystems. Usually, the definition of specialization is deceptively complex, and the quantification of specialists' and generalists' needs to be based on different abiotic and habitat conditions (Denelle et al., 2020; Devictor et al., 2010). We examined an alternative method for classifying specialists and generalists based on the number of mutualistic links of species. Compared with traditional methods, mutualistic links have distinct advantages in distinguishing between habitat specialists and generalists, and researchers urgently need an efficient way to distinguish specialists and generalists using interactions between species (Blüthgen et al., 2006; Zografou et al., 2020). In particular, given an interacting ecosystem, we can easily derive a two‐layer network with two collective abundances according to the mutualistic link: one consisting of generalist species and the other consisting of specialist species.

We observed the composition of generalists and specialists in real ecosystems and proposed that the decline of generalists leads to local ecosystem collapse, while the decline of specialists rarely leads to system collapse. Our study confirms recent empirical and theoretical findings (Chacoff et al., 2018; Zografou et al., 2020) that ecosystems consist of a reliable core of generalists, accompanied by a changing suite of specialists. Furthermore, we applied disturbance to our two‐layer network and the results showed that the sub‐networks of ecological specialists and generalists have properties similar to those of the global network. Our two‐layer network can serve as an effective tool for observing global dynamics in real ecosystems.

Overall, our work provides a reliable tool for specialists' and generalists' research in multiple interactions. In particular, we supplement the research of identifying specialists and generalists in ecosystems through the mutualistic interaction of species. Our work can serve as the basis for extension to other research, such as the invasion of specialists or generalists, the tolerance of specialists or generalists to global environmental change, etc. Furthermore, we emphasize that simple subsets of local ecosystems may feed back important information about ecosystem function and status.

## AUTHOR CONTRIBUTIONS

**Dongli Duan:** Conceptualization (equal); funding acquisition (lead); methodology (equal); resources (equal); writing – review and editing (equal). **Jiale Hang:** Conceptualization (lead); data curation (equal); formal analysis (equal); methodology (equal); validation (equal); writing – original draft (lead). **Chengxing Wu:** Conceptualization (equal); data curation (equal); formal analysis (equal); software (equal); writing – original draft (equal). **Xue Bai:** Writing – review and editing (supporting). **Yisheng Rong:** Writing – review and editing (supporting). **Gege Hou:** Writing – review and editing (supporting).

## CONFLICT OF INTEREST STATEMENT

The authors declare no conflicts of interest.

## Data Availability

Data were obtained from real plant‐pollinator networks in the web‐of‐life database (www.web‐of‐life.es).
